# Substitutions in conserved regions preceding and within the linker affect activity and flexibility of tRNase Z^L^, the long form of tRNase Z

**DOI:** 10.1371/journal.pone.0186277

**Published:** 2017-10-18

**Authors:** Makenzie Saoura, Kyla Pinnock, Maria Pujantell-Graell, Louis Levinger

**Affiliations:** York College of The City University of New York, Jamaica, New York, United States of America; Max-Planck-Institut fur terrestrische Mikrobiologie, GERMANY

## Abstract

The enzyme tRNase Z, a member of the metallo-β-lactamase family, endonucleolytically removes 3’ trailers from precursor tRNAs, preparing them for CCA addition and aminoacylation. The short form of tRNase Z, tRNase Z^S^, functions as a homodimer and is found in all prokaryotes and some eukaryotes. The long form, tRNase Z^L^, related to tRNase Z^S^ through tandem duplication and found only in eukaryotes, possesses ~2,000-fold greater catalytic efficiency than tRNase Z^S^. tRNase Z^L^ consists of related but diverged amino and carboxy domains connected by a flexible linker (also referred to as a flexible tether) and functions as a monomer. The amino domain retains the flexible arm responsible for substrate recognition and binding while the carboxy domain retains the active site. The linker region was explored by Ala-scanning through two conserved regions of *D*. *melanogaster* tRNase Z: N_dom_T_prox_, located at the carboxy end of the amino domain proximal to the linker, and T_flex_, a flexible site in the linker. Periodic substitutions in a hydrophobic patch (F_329_ and L_332_) at the carboxy end of N_dom_T_prox_ show 2,700 and 670-fold impairment relative to wild type, respectively, accompanied by reduced linker flexibility at N-T inside the N_dom_- linker boundary. The Ala substitution for N_378_ in the T_flex_ region has 10-fold higher catalytic efficiency than wild type and locally decreased flexibility, while the Ala substitution at R_382_ reduces catalytic efficiency ~50-fold. These changes in pre-tRNA processing kinetics and protein flexibility are interpreted in light of a recent crystal structure for *S*. *cerevisiae* tRNase Z, suggesting transmission of local changes in hydrophobicity into the skeleton of the amino domain.

## Introduction

Transfer RNA (tRNA) is central to translation [1]. Sequencing of the first tRNAs established a canonical secondary structure (cloverleaf) which arises from intramolecular base pairing, and a conserved L-shaped tertiary structure. D—T loop pairing forms the elbow, with the anticodon and acceptor stem at opposite ends. CCA at the 3’ end is universally conserved. C_74_C_75_ of P-site tRNA H-bond with large subunit rRNA, positioning it for peptidyl transfer; the 2’OH of A_76_ on the peptidyl tRNA participates critically in catalysis.

tRNAs are transcribed as precursors and processed by endonucleolytic removal of a 5’ leader by RNase P, first characterized as a ribozyme and later shown to be a protein-only enzyme in mitochondria and chloroplasts (for reviews, see [2], [3]). Some tRNAs are transcribed with introns that are removed by splicing and all tRNAs undergo extensive post-transcriptional nucleoside modification. The 3’ trailer is removed by a combination of endo- and exonucleases in *E*. *coli*, in which -CCA_76_ is transcriptionally encoded. In eukaryotes, -CCA_76_ is not transcriptionally encoded; CCA-addition is thus required in eukaryotic nuclei and plastids. tRNase Z provides the principal mechanism for endonucleolytic removal of eukaryotic 3’ trailers, leaving the discriminator base (N_73_) with a 3’-OH ready for CCA addition. This pathway may be complemented by exonucleases in *S*. *cerevisiae* ([4], [5]).

The tRNase Z function and a gene encoding the enzyme are widely conserved [6]. A short and long form (tRNase Z^S^ and tRNase Z^L^, respectively) both endonucleolytically cleave pre-tRNA 3’ end trailers, however the two forms are unevenly distributed among the domains of life. Bacteria and archae exclusively possess tRNase Z^S^. While tRNase Z^S^ is found in some eukaryotes, tRNase Z^L^ is more widespread (for example, tRNase ZS is absent from *S*. *cerevisiae*, *C*. *elegans* and *D*. *melanogaster*).

tRNase Z is a member of the β-lactamase family of metal-dependent hydrolases, characterized by an αβ/βα sandwich fold with the active site located at the interface between the domains [7]. Motifs I–V are conserved, including seven residues (His and Asp) that coordinate binding of two Zn^++^ ions which direct H_2_O in general in-line acid-base catalysis, four of them in the signature His cluster (HxHxDH; Motif II).

tRNase Z^S^ functions as a homodimer of identical subunits. In addition to Motifs I–V, a unique flexible arm [8], [9] protrudes from the globular core of tRNase Z and binds the elbow of tRNA, directing the acceptor stem including the scissile bond into the active site of the enzyme. The flexible arm in subunit 1 thus positions the 3’ end of the substrate in the active site of subunit 2.

Sequence and structural studies show that tRNase Z^L^ emerged as a tandem duplication of tRNase Z^S^ with subsequent divergence of the amino and carboxy domains (first suggested by [10] and subsequently supported by numerous studies, reviewed in [11]). The amino domain retained the flexible arm but lost the key His and Asp residues from the catalytically important motifs otherwise related by sequence, while the carboxy domain retained functional motifs required for catalysis and lost the flexible arm. The resulting enzyme is better adapted for pre-tRNA 3’ end processing, based on ~2,000-fold higher catalytic efficiency of *H*. *sapiens* tRNase Z^L^ than that of tRNase Z^S^ [12]. tRNase Z^L^ is a monomer in solution based on size exclusion chromatography [13] and in the recently solved crystal structure of *S*. *cerevisiae* tRNase Z [11] (a tRNase Z^L^).

A 62–85 residue flexible linker joins the conserved, relatively stable amino and carboxy domains in tRNase Z^L^ [13]. The boundary between the linker and the carboxy domain is delineated by homology between the carboxy domain of tRNase Z^L^ and the amino end sequence of tRNase Z^S^. Interestingly, the linker spans the protein surface like a flexible strap [11]; the interface between the amino and carboxy domains of tRNase Z^L^ is much like the dimer interface of tRNase Z^S^.

Within the amino domain of tRNase Z^L^, sequences align with the carboxy domain of tRNase Z^L^ and with tRNase Z^S^ up to and including the flexible arm. In the second half of the amino domain, homology blocks identifiable in tRNase Z^L^s are less clearly related to sequences in the carboxy domain.

We used previously developed methods ([14]; [13]) to investigate the function and flexibility in regions preceding and within the linker. Ala scans (substitution of alanine for each wild type residue) with processing kinetics were performed followed by flexibility analysis of selected variants. Results were interpreted based on local changes in hydrophobicity in light of the newly available *S*. *cerevisiae* tRNase Z structure [11].

## Methods

### Structure modeling

Secondary structure prediction was performed using PsiPred. Hydropathy plots were obtained using the Wolfenden subprogram [15] in ExPASy. The 1^st^ inframe methionine (MYLV…) of *D*. *melanogaster* tRNase Z (NCBI NP_724916.1) and the following 19 residues are interpreted to be a mitochondrial targeting sequence [16] and the nuclear form (presented here) is numbered from the 2^nd^ inframe methionine (…MAAT…). The recently published *S*. *cerevisiae* tRNase Z structure [11] was interpreted using PyMOL [17].

### Ala scanning mutagenesis

Conserved regions were selected for Ala scanning mutagenesis, one just before the flexible linker and two within the linker. N_dom_T_prox_ consists of 19 residues in the last homology block in the amino domain on the amino side of the linker (H315 –G_333_). T_flex_ consists of 9 residues from the most flexible conserved internal region of the linker (M_376_ –R_384_). The PEEY region, glutamate rich and less conserved, consists of 9 residues further toward the carboxy end of the linker (P_397_—H_405_). These 37 residues were individually substituted with alanine by replacing the wild type codon at each position with a GCC triplet using A, B amplification and A-B segment joining by PCR and overlap extension PCR, as previously described [18]. ~40-mer oligonucleotides were typically used with the 1, 2 or 3 nt substitution in the middle and with a GC-rich cluster at the 3’ end for stability of primer annealing. The AflII site (nt 1077–1082) subcloning forward primer combined with the reverse mutagenesis primer were used to amplify the A segment using a wild type template. The coding strand (forward) mutagenesis primer combined with the SacI site (nt # 1527–1532) subcloning reverse primer were used to amplify the B segment. A and B segments were gel purified and joined by overlap extension and amplification using the AflII forward and SacI reverse primers. Joined segments were gel purified, recovered, double digested, recovered, and ligated into the AflII-SacI digested vector from which the 454 bp wild type segment had been removed. Plasmids that passed the RE screen were sequenced (Macrogen) to confirm presence of each intended GCC codon and absence of any other sequence changes. The FastBacHT (Invitrogen) transfer vectors with variant tRNase Z cDNAs were transposed into bacmids using DH10Bac (Invitrogen). Large true white colonies produced by successful transformation and transposition were selected for bacmid DNA isolation and transfection into insect Sf9 cells using Cellfectin 2 reagent (Invitrogen).

### Baculovirus expression and affinity purification

Amplified baculoviruses with variant *D*. *melanogaster* tRNase Z cDNAs were used to infect insect Sf9 cells for 72 h using Hyclone SFX insect cell medium supplemented with 0.5% FBS to minimize degradation of recombinant proteins by endogenous proteases. Cells were lysed with NP40, expressed proteins were affinity purified using Ni-NTA Sepharose (Qiagen) and the 6XHis tag was cleaved overnight at 4°C with AcTEV protease as previously described [18].

### tRNase Z reaction kinetics

Nuclear encoded pre-tRNA^Arg^ transcript was prepared with T7 RNA polymerase and cleaved using a cis-acting hammerhead leaving a 5’-OH at +1 of the tRNA as previously described [19]. Kinasing with γ-32P-ATP by polynucleotide kinase was performed at +1 of the tRNAs, followed by gel purification and recovery. The processing reaction buffer (PB) consisted of 25 mM Tris-Cl pH 7.2, 2.5 mM MgCl_2_, 1 mM freshly prepared DTT, and 100 μg/ml BSA. Unlabeled substrate concentration was varied over a range of 4–100 nM with a fixed trace amount of 5’-labeled substrate. tRNase Z stocks were adjusted to 25 μM before use from which a dilution series was prepared. Analytical lanes were run with known concentration standards for both input tRNase Z and unlabeled tRNA and the enzyme and unlabeled substrate concentrations used in each experiment was corrected accordingly. Reactions at 28°C were sampled after 5, 10 and 15 min, and quenched with formamide-marker dye mix on ice. Electrophoresis of the samples was carried out on a 6% polyacrylamide gel containing 8 M urea. Gels were dried and exposed overnight using a phosphor screen, which was scanned using a Typhoon 9410 imager and analyzed with IQTL v8.1. Each lane trace yielded a % product and the time course results were converted to % product/min using Excel, equivalent to 0.01 X V/[S], then converted to V X 10^−11^ M/min by multiplying by nM [S], and further analyzed using the single ligand binding function in SigmaPlot. *k*_*cat*_ was obtained by dividing V_max_ by [E]. Concentration of each variant enzyme was adjusted as necessary depending on the impairment factor observed in previous kinetic experiments. The processing experiments with each variant were repeated until acceptable standard errors were achieved.

### Flexibility of wild type and variant tRNase Z analyzed by limited proteolysis and protein electrophoresis

Wild type and selected variant tRNase Zs were proteolyzed with trypsin at 1 μg/ml in PB at 28°C and reactions were sampled after 0, 3, 10 and 30 min reaction. Limited proteolysis reactions were analyzed on 1D SDS polyacrylamide gels or using a 2D system (BioRad) with isoelectric focusing in 0.75 mm diam 1^st^ dimension tube gels and SDS electrophoresis in the 2^nd^ dimension as previously described [13]. Protein bands and spots were detected by staining with Sypro Orange and scanning with a Typhoon 9410 and quantitated using IQTLv8.1.

## Results

**Fig 1:**
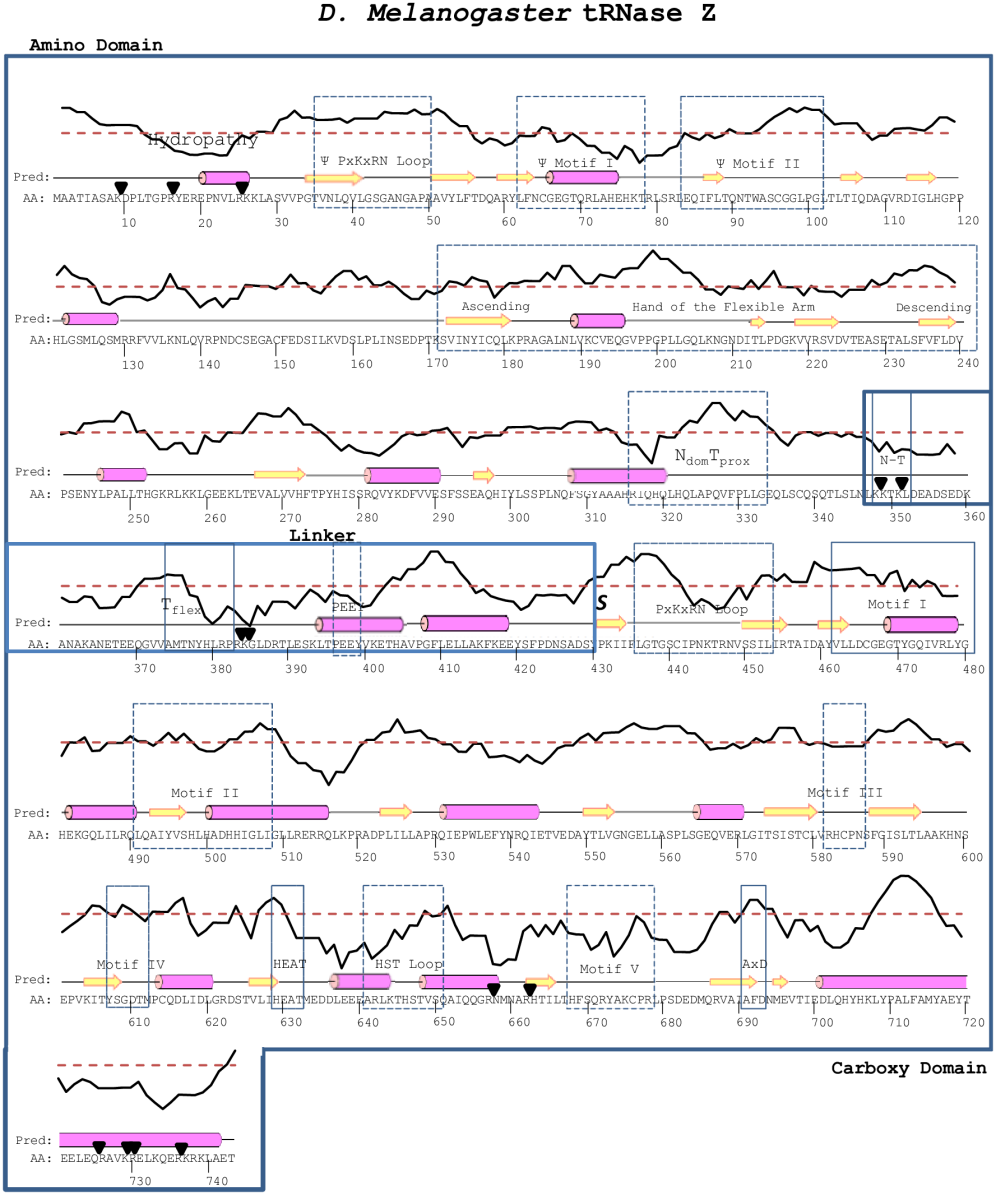
*D*. *melanogaster* tRNase Z primary structure and prediction of secondary structure and hydrophobicity. The amino acid sequence is shown with secondary structure predicted by PsiPred. Rectangles enclose the amino and carboxy domains joined by the flexible linker; dashed lines indicate conserved motifs and black triangles indicate identified trypsin cleavage sites [13] which occur at flexible, hydrophilic regions. Directly above the predicted secondary structure, a hydropathy plot (created with ExPASy using the Wolfenden scale [15]) depicts the relative hydrophobic and hydrophilic character of the corresponding regions, the dashed red line indicating approximate neutrality.

A local hydropathy plot [15] provides a useful extension to PsiPred for interpretation of tRNase Z structure and flexibility (Fig 1). For example, pronounced hydrophobicity troughs found close to both ends of the protein, typical of globular proteins in aqueous solution, coincide with flexible regions (cf [13]). N-T and T_flex_, the two most flexible regions in the linker, are also predicted local hydrophobicity troughs.

**Fig 2:**
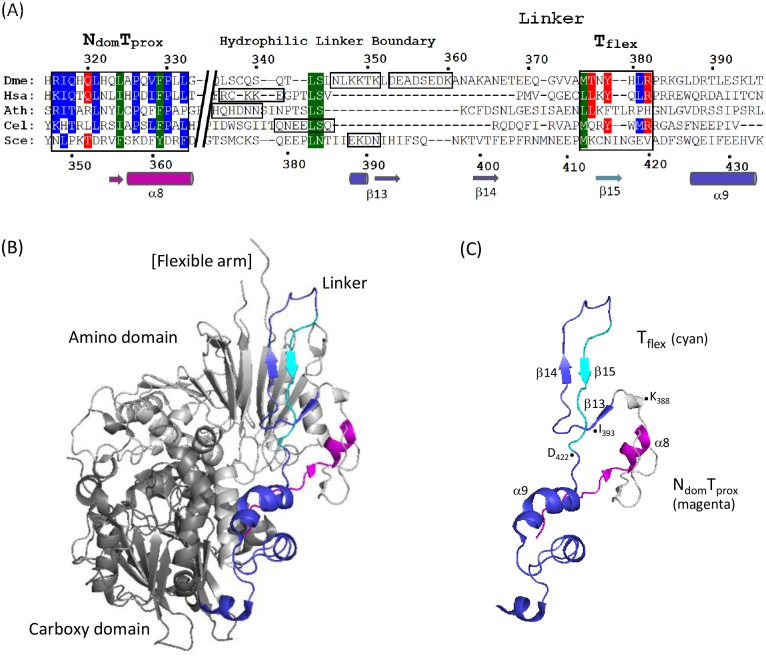
The flexible linker of tRNase Z^L^. The flexible linker of tRNase Z^L^ connects the enzyme’s amino (binding) and carboxy (catalytic) domains. (A) Multiple sequence alignment includes a conserved region directly preceding the linker as well as within the linker of tRNase Z^L^. Sequences are from *Drosophila melanogaster* (NP_724916.1), *Homo sapiens* (NP_060597.4), *Arabidopsis thalania* (NP_188247.2), *Caenorhabditis elegans* (NP_001023109.1), and *Saccharomyces cerevisiae* (NP_013005.1). Residue numbers for *D*. *melanogaster* tRNase Z are shown above and for *S*. *cerevisiae* below. Secondary structure elements identified in [11] in *S*. *cerevisiae* tRNase Z are shown below. (B) An overview of the crystal structure of *S*. *cerevisiae* TrZ1 ([11]; PDB 5MTZ) shown in cartoon using PyMOL. The linker (shown in blue and cyan) runs like a strap along the enzyme’s exterior, extending from the amino domain (light grey) to the carboxy domain (dark grey). (C) N_dom_T_prox_ and linker shown in isolation.

The carboxy domain of tRNase Z^L^ is homologous to tRNase Z^S^, including the active site. Similarly, the flexible arm (FA) in tRNase Z^L^ is related to one of the three branches of flexible arms [9], and the sequence that precedes it is also related, in agreement with the evolution of tRNase Z^L^ from a tandem duplication of tRNase Z^S^ followed by divergence of the amino and carboxy domains. Less is known about the flexible linker of tRNase Z^L^, however. The *S*. *cerevisiae* tRNase Z linker closely follows the exterior contours of the protein as it joins the amino and carboxy domains ([11]; Fig 2). A multiple sequence alignment (Fig 2A; see [20]) combined with the flexibility results ([13]; Fig 1) suggest the most important regions for further investigation of this extrinsic feature of the enzyme.

Little structural information was available on the amino domain and linker until the recent publication of a *S*. *cerevisiae* tRNase Z structure [11]. The basic structure of the *S*. *cerevisiae* tRNase Z amino domain is an αβ/βα sandwich fold, like that of the carboxy domain. The flexible arm, located between two strands of β twisted sheet, is extruded from the body of the amino domain. The tRNase Z linker spans the globular core of the enzyme like a strap (Fig 2B; [11]). The linker is an adjunct to, not a substitute for, the domain interface between the amino and carboxy domains, which is much like that observed in the tRNase Z^S^ homodimer ([11]; cf [8]).

N_dom_T_prox_ (within the N domain, proximal to the linker) is the last such homology block preceding the linker [19], [13]. Based on the *S*. *cerevisiae* tRNase Z crystal structure [11], the 1^st^ half of N_dom_T_prox_ has little secondary structure, followed by a short β strand and an α helix (α8) with high local hydrophobicity. A flexible hydrophilic patch located on the linker side of the amino domain—linker boundary designated N-T, less conserved than N_dom_T_prox_, which in *S*. *cerevisiae* tRNase Z consists of a short helix followed by a β strand (β13), gives rise to the limited proteolysis species C_dom_1 [13]. Another conserved flexible hydrophilic region designated T_flex_, found ~35 residues within the linker, gives rise to the C_dom_2 family of proteolysis products.

The regions subjected to single residue Ala substitution and kinetic analysis include 19 residues in N_dom_T_prox_ and 13 residues in T_flex_. A short sequence further into the linker is characterized by contiguous glutamates (PEEY region). The goal of an Ala scan is to discover residues of sufficient importance that, when replaced by Ala, cause a significant effect on enzyme activity. Such effects were not observed within the PEEY region, which will therefore not be discussed further. The N-T region is generally conserved in location and hydrophilicity but does not align well and was therefore not examined. Once results of processing kinetics were available, flexibility of selected variants with suggestive functional impairments were studied by limited proteolysis with trypsin and protein gel electrophoresis as in [13].

### Substitutions in two bulky hydrophobic N_dom_T_prox_ residues close to the N_dom_-linker boundary greatly impair processing and also reduce flexibility in the N-T region

**Fig 3:**
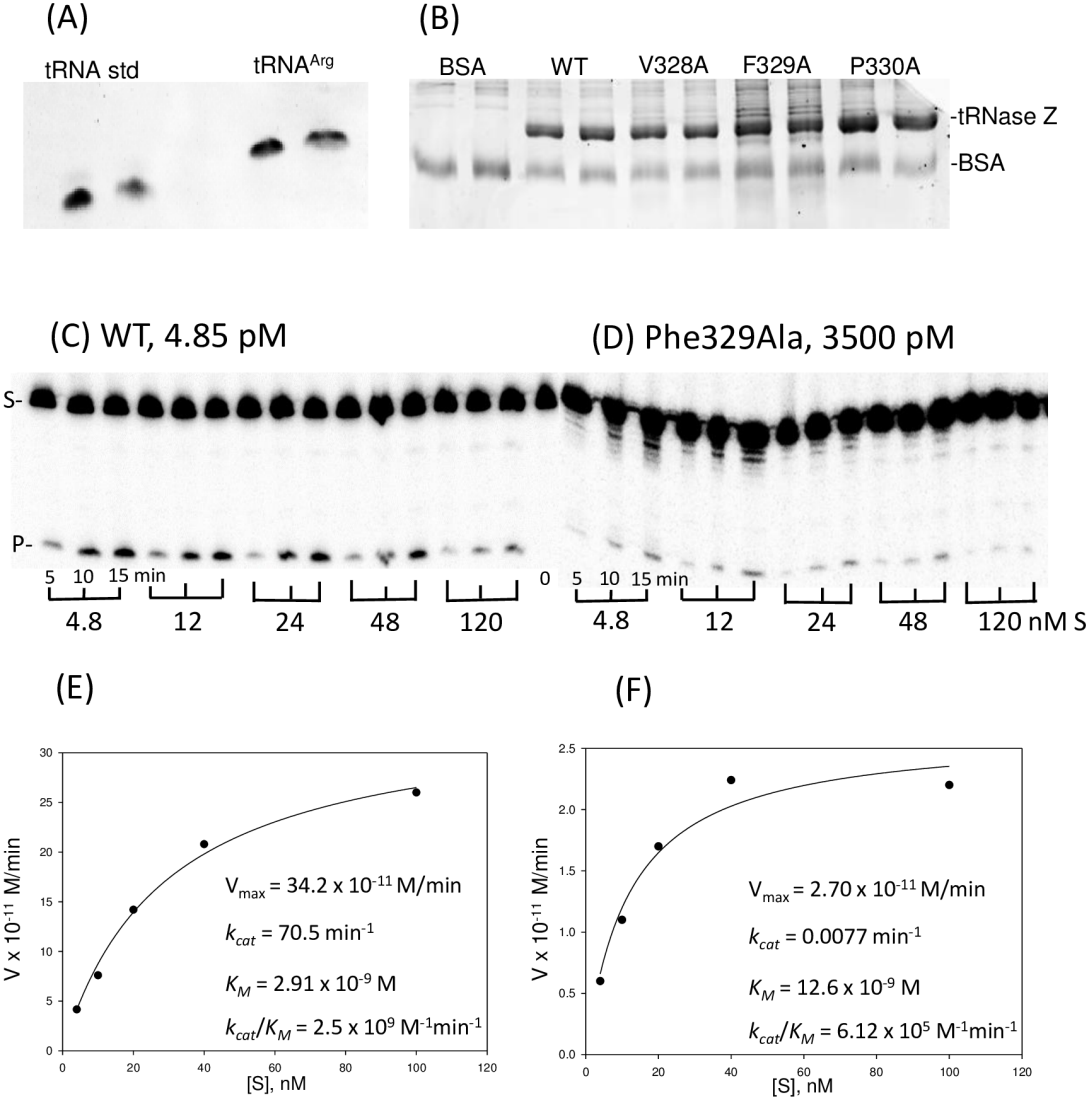
Processing kinetics with wild type tRNase Z and Phe329Ala variant. The Phe329Ala substitution impairs pre-tRNA^Arg^ processing approximately 2,700-fold compared to WT. (A) Enzymes expressed using baculovirus, affinity purified and used for processing experiments were analyzed with 10% polyacrylamide gel, here shown with two other alanine substitutions from the N_dom_T_prox_ region, to correct the final enzyme concentrations used in the kinetic experiments. (B) The concentration of unlabeled pre-tRNA^Arg^ substrate used in a substrate concentration series was determined using A_260_ readings by NanoDrop, and confirmed or corrected by comparison with a eukaryotic tRNA standard on a 6% gel. (C-D) Michaelis-Menten kinetics experiments were performed using constant concentration of ^32^P labeled substrate with added unlabeled substrate varied over a concentration range from 4.8–120 nM (shown below gel panels), with reactions incubated at 28°C and sampled after 5, 10, and 15 minutes. WT and F329A enzyme concentrations (shown above gel panels) were adjusted to obtain roughly equivalent product in the variant, here requiring an almost 1,000-fold higher concentration of F329A variant than WT. (E-F) Michaelis-Menten plots with kinetic parameters calculated using SigmaPlot. The 2,700X decrease in catalytic efficiency for the Phe329Ala variant is principally due to a 1,000-fold decrease in *k*_*cat*_, combined with a modest increase in *K*_*M*_.

**Fig 4:**
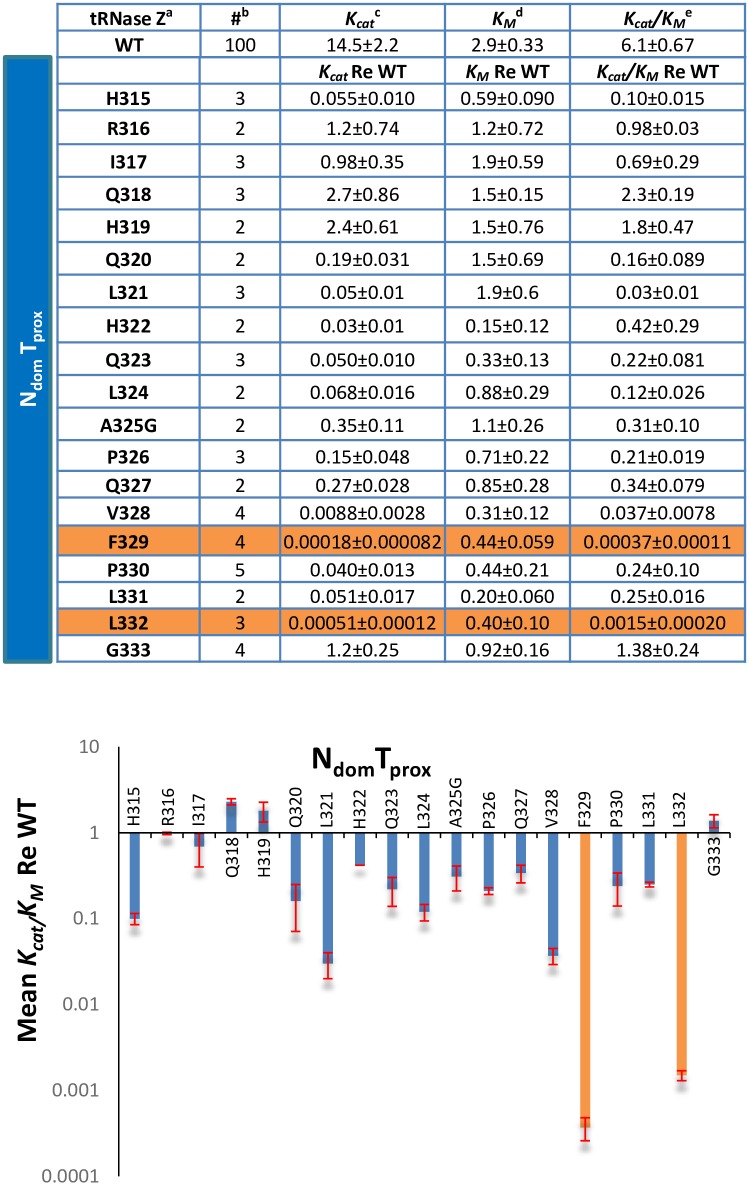
Tabulated variant kinetics for the Ala scan through the N_dom_T_prox_ region. Means and standard errors of Michaelis-Menten experiments with tRNase Z processing of pre-tRNA^Arg^. Kinetic parameters re: WT are shown for each variant, calculated using the data from a WT experiment run in tandem the same day and then averaged. ^a^The form of tRNase Z (WT or Variant), ^b^The number of times experiment was repeated, ^c^min^-1^, ^d^x10^-8^ M, ^e^x10^8^ M^-1^min^-1^. The bar graph below shows values from the table above.

The Ala scan processing results in the 1^st^ half of N_dom_T_prox_, suggested by PsiPred to be in α-helix, are unremarkable. Alanine substitutions in two bulky hydrophobics spaced three residues apart close to the carboxy end of N_dom_T_prox_, Phe329Ala and Leu332Ala, strikingly impair processing with impairment factors of 2,700X and 700X relative to wild type (Figs 3 and 4). In the example illustrated (Fig 3), it was necessary to use the Phe329Ala variant at a >1,000-fold higher concentration than wild type enzyme to obtain a comparable series of processing time courses over the range of unlabeled substrate concentrations used in kinetic experiments.

**Fig 5:**
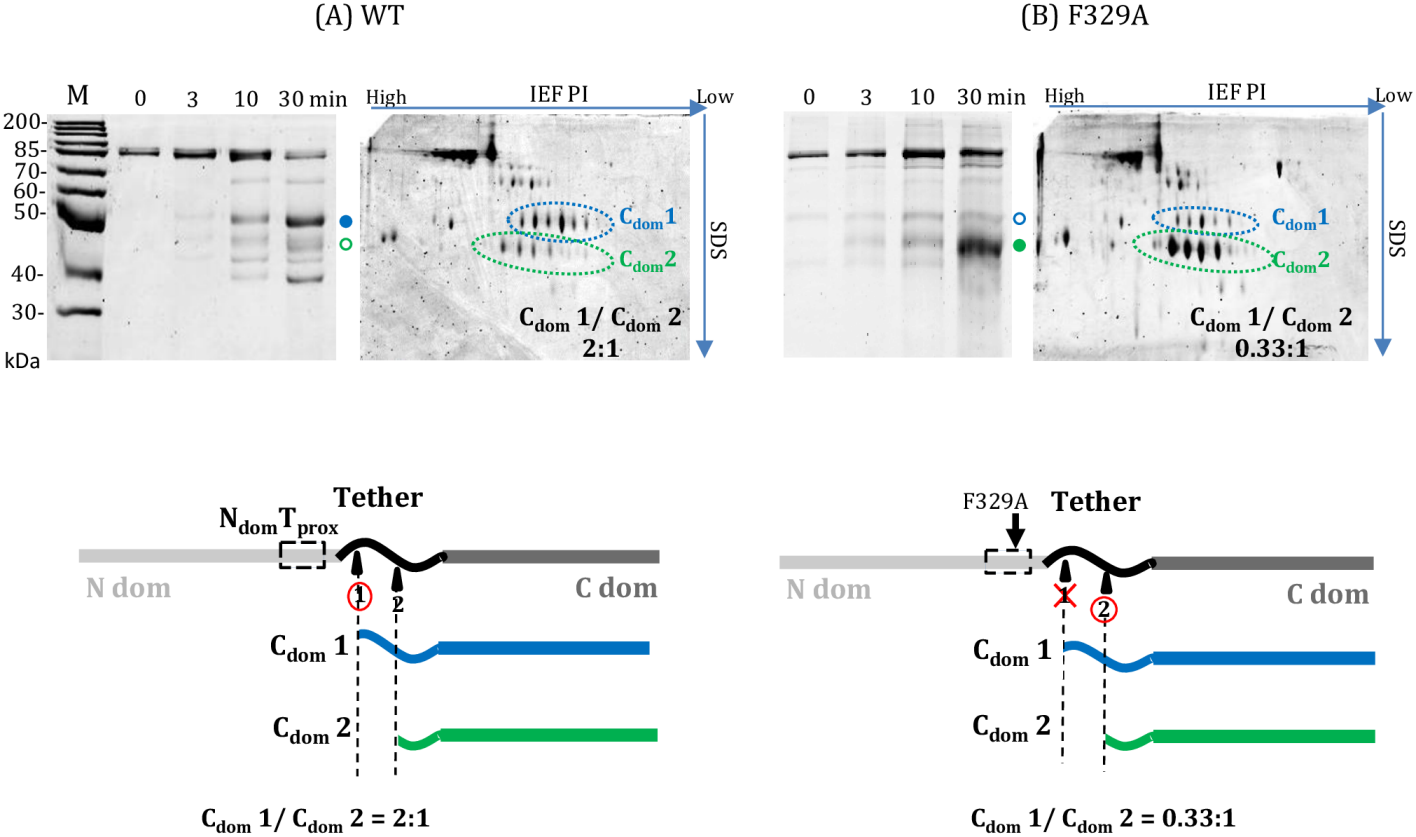
*D*. *melanogaster* tRNase Z Phe329Ala substitution in the N_dom_T_prox_ region locally reduces flexibility. A) WT; B) Phe329Ala variant. Trypsin digestions were sampled at 0, 3, 10, and 30 minutes and electrophoresed on 1D SDS gels (left panels in A and B). Additionally, 10 minute reactions were electrophoresed using 1^st^ dimension isoelectric focusing followed by 2^nd^ dimension SDS-PAGE (right panels in A and B). C_dom_1 species are marked in blue (• on the 1D SDS-PAGE and enclosed in a dashed ellipse in the accompanying 2D gel); C_dom_2 species in green (• on the 1d SDS-PAGE and enclosed by a green dashed ellipse in the 2D gel). Cleavage at clusters of basic residues accounts for the multiple C_dom_1 and C_dom_2 species. C_dom_1/C_dom_2 ratios determined with IQTL are shown at lower right of the 2D gel panels in A, B. The schematic diagrams below illustrate the location of N_dom_T_prox_ with respect to the N-T and T_flex_ sites.

These substitutions for bulky hydrophobic residues on the carboxy side of N_dom_T_prox_ were selected for further examination for limited proteolysis with trypsin and protein gel electrophoresis (Fig 5 and data not shown). Phe329Ala demonstrated a marked change in the ratio of stable C_dom_ products produced upon trypsin cleavage compared to WT tRNase Z (similar results were obtained from Leu332Ala, not shown). The N_dom_T_prox_ region is proximal to the preferred trypsin N-T cleavage site at K_348_/K_351_ which produces stable C_dom_1species (accompanying schematics at bottom of Fig 5) that differ slightly in size and charge depending on cleavage at clustered basic residues ([14]; Fig 1). The T_flex_ site further into the linker at R_384_/K_385_ gives rise to the smaller C_dom_2 species. The C_dom_1 to C_dom_2 ratio in WT tRNase Z is 2:1; in the F_329_ variant this ratio decreases to 0.33:1, showing that the alanine substitution at F_329_ locally reduces N-T site flexibility.

### Effects of T_flex_ region substitutions on processing kinetics and local flexibility

**Fig 6:**
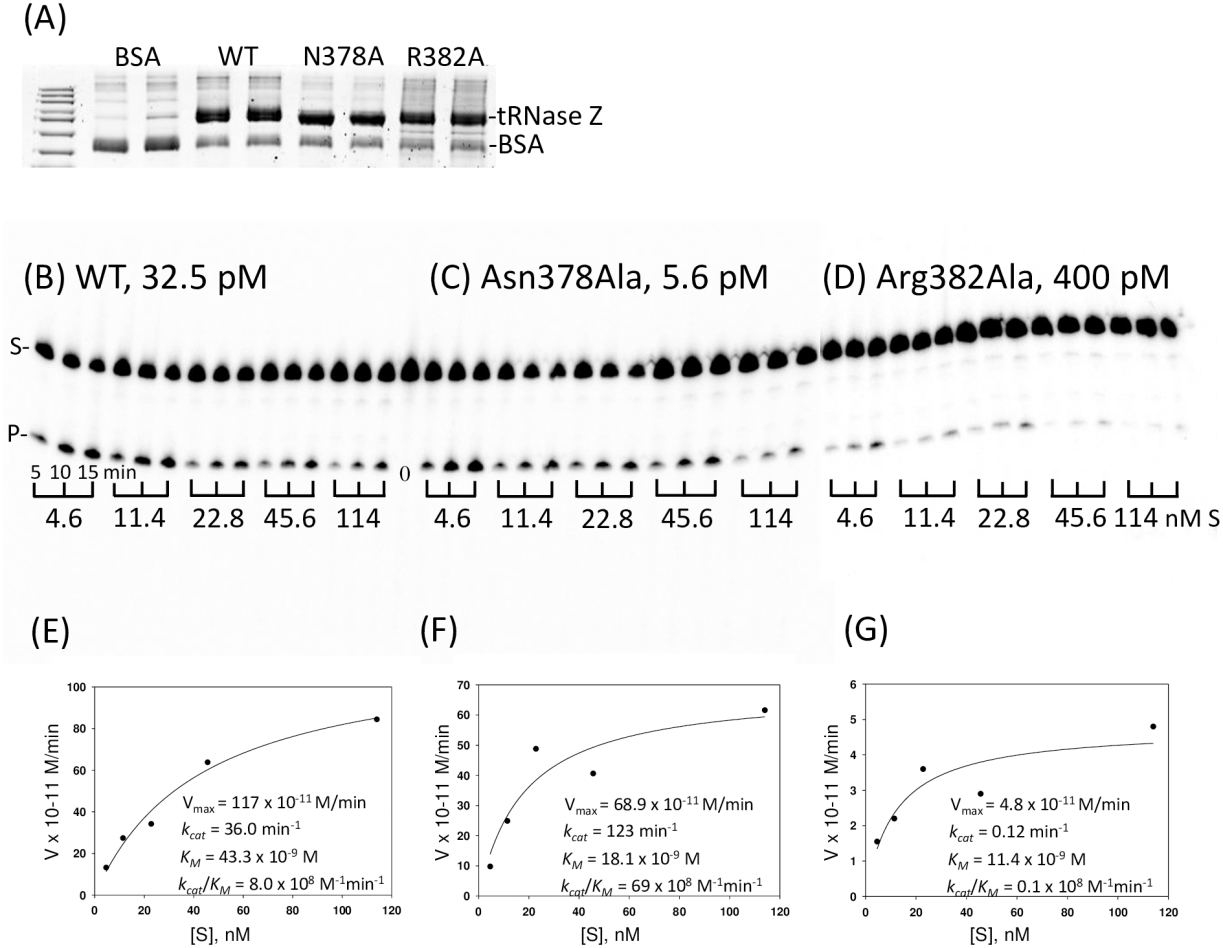
Processing kinetics with wild type tRNase Z and the T_flex_ variants Asn378Ala and Arg382Ala. The Asn378Ala substitution increases processing efficiency, while the Arg382Ala substitution impairs processing of pre-tRNA^Arg^. (A) tRNase Z dilutions used in processing experiments were electrophoresed on a 10% polyacrylamide SDS gel and compared to a BSA standard to determine concentrations. (B-D) Kinetic experiments were performed with a constant concentration of 5’ end ^32^P labeled pre-tRNA^Arg^ substrate and varying concentration of unlabeled substrate, from 4.6–114 nM as indicated below gel panels. Reactions were sampled after 5, 10, and 15 minute incubation at 28°C. Wild type enzyme was used at 32.5 pM; N378A enzyme at 5.6 pM, and R382A enzyme at 400 pM (above gel panels). Phosphorimages were obtained using a Typhoon 9410 scanner. % product/minute, equivalent to V/[S], was determined using IQTLv8.1 software. (E-G) Michaelis-Menten plots were created using SigmaPlot, with kinetic parameters displayed on the corresponding graphs.

**Fig 7:**
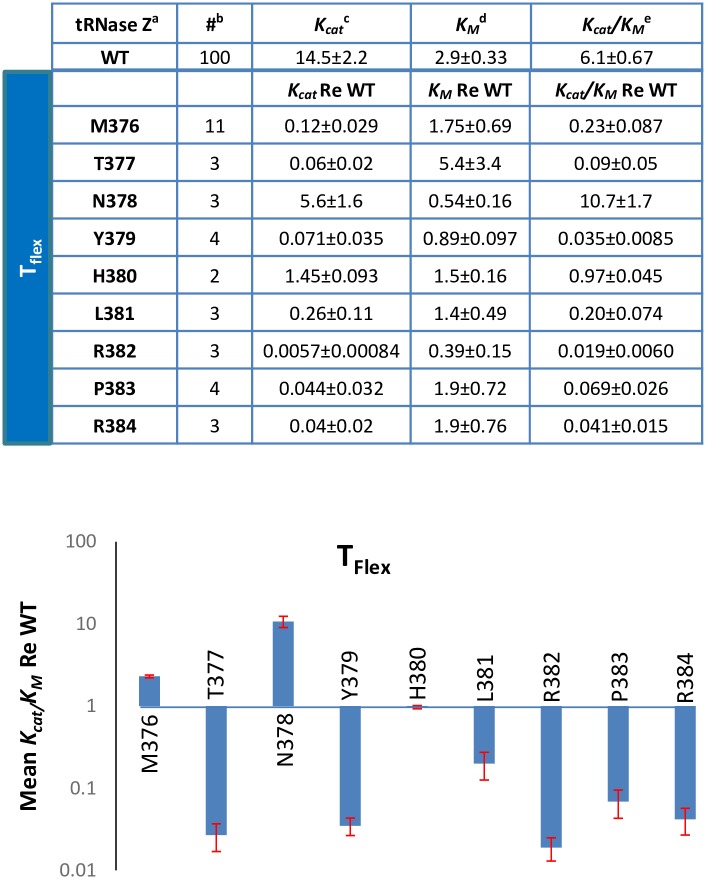
Compilation of T_flex_ variant kinetic results. Means and standard errors of Michaelis-Menten experiments with tRNase Z processing of pre-tRNA^Arg^ using Ala substitution variants in the T_flex_ region. Kinetic parameters re: WT are shown for each variant. The ratios were calculated using data from a WT experiment run in tandem the same day before being averaged. ^a^The form of tRNase Z (WT or Variant), ^b^The number of times experiment was repeated, ^c^min^-1^, ^d^x10^-8^ M, ^e^x10^8^ M^-1^min^-1^. The bar graph below shows the results from the table above.

**Fig 8:**
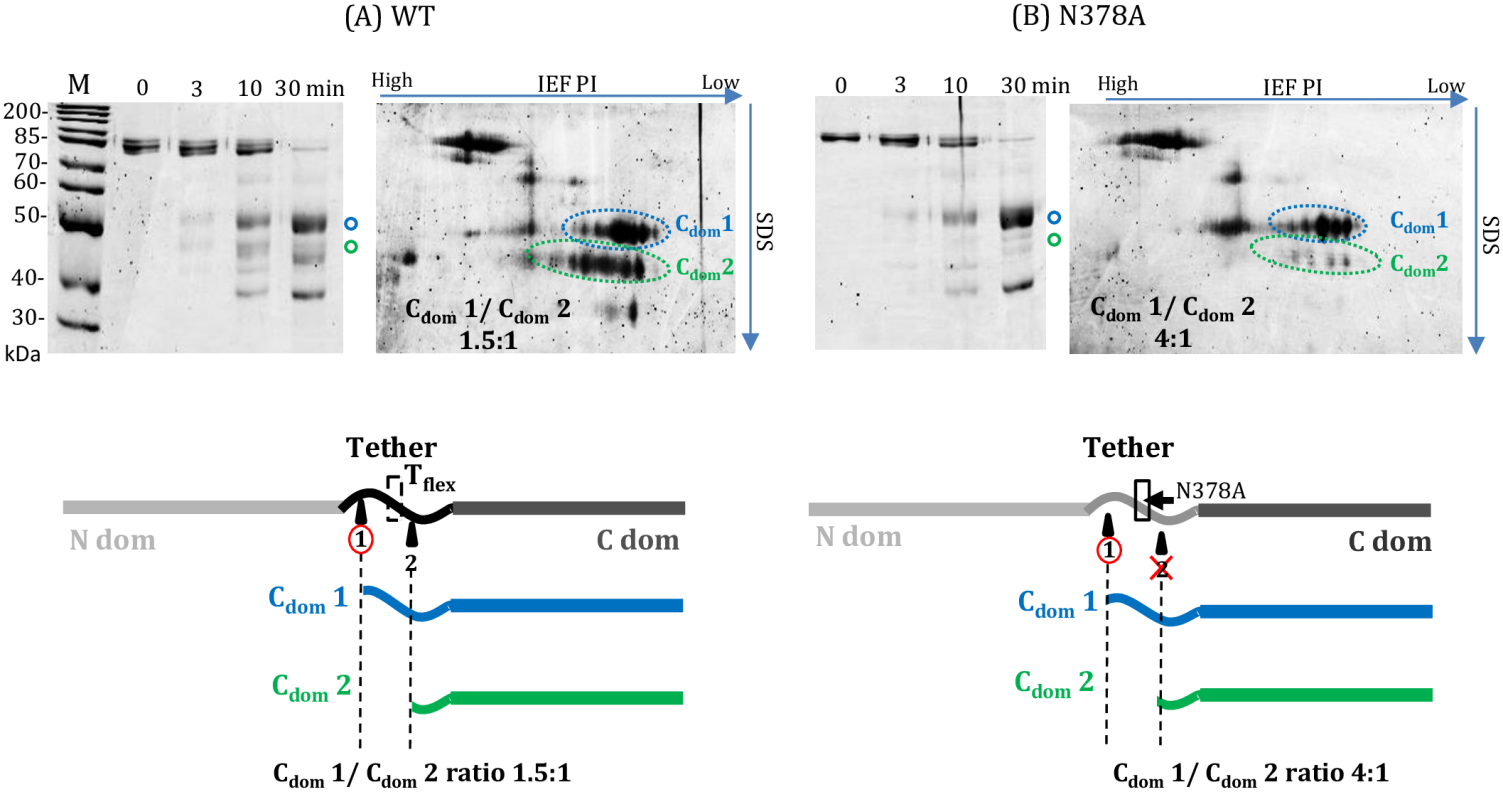
*D*. *melanogaster* tRNase Z variant Asn378Ala reduces local flexibility. Trypsin digestions and electrophoresis were performed as described in Fig 5 and Methods. (A) WT enzyme; (B) tRNase Z with the N378A substitution. Color coding of spots and bands are the same as in Fig 5. The N378A substitution dramatically reduces flexibility at the nearby T_flex_ site relative to WT.

Of the nine T_flex_ alanine variants expressed and analyzed with processing kinetics, Arg382Ala at the carboxy end of the T_flex_ region showed the greatest impairment factor, an approximately 50X reduced processing efficiency relative to WT tRNase Z (Fig 6). Multiple sequence alignment shows this to be a conserved residue (Fig 2). Asn378Ala, a substitution in a non-conserved residue near the amino boundary of T_flex_, unexpectedly showed a tenfold increase in processing efficiency (Figs 6 and 7). Additionally, the Asn378Ala substitution markedly reduces local flexibility as shown by limited proteolysis (Fig 8). The T_flex_ region includes the trypsin cleavage site at R_384_/K_385_ which gives rise to the stable C_dom_2 species. In WT tRNase Z the spot intensity ratio of C_dom_1 to C_dom_2 is 1.5:1 (from schematic at bottom of Fig 8, like the value obtained in Fig 5). For the Asn378Ala variant this ratio increases to 4:1. Alanine substitution at N_378_ thus causes a dramatic decrease in C_dom_2 species seen after trypsin digestion due to a local decrease in flexibility.

### A subdomain defined by interior hydrophobicity arises from interactions across the amino domain—Flexible linker boundary

**Fig 9:**
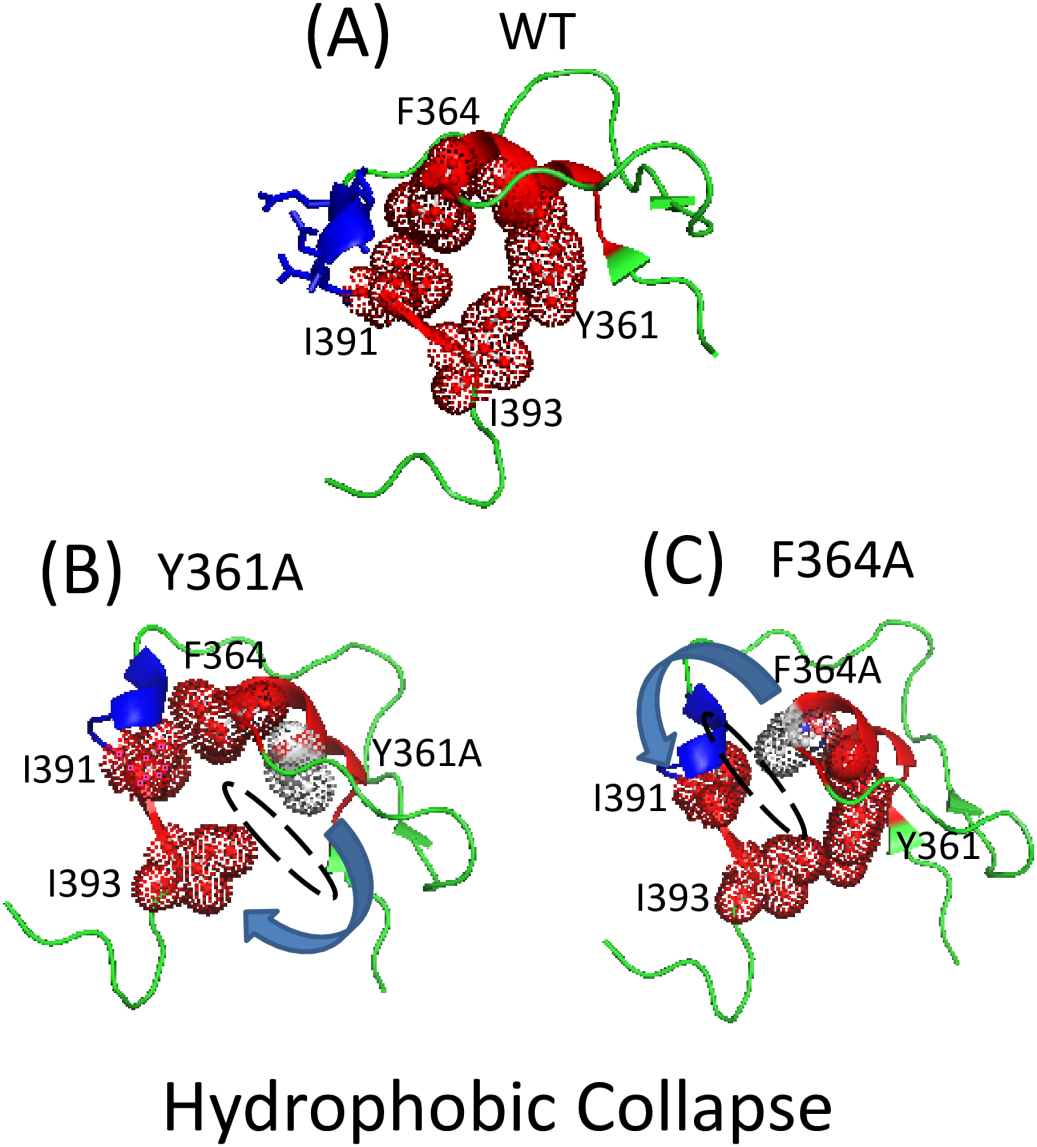
A cluster formed by hydrophobic interactions between residues in N_dom_T_prox_ and N-T. A) The region of *S*. *cerevisiae* tRNase Z ([11]; 5MTZ) from α8 through β13 is shown in cartoon using PyMOL. α8 and β13 are in red and a short helical hydrophilic segment preceding β13 (E_387_KDN) is in blue with sticks. Key hydrophobic residues in α8 and β13 are shown in ball and stick with dots. (B) Y_361_ is substituted with Alanine (white); (C) F_364_ is substituted with Alanine. The substitutions in (B, C) model the substitution of the smaller R-group of Alanine for the bulky hydrophobic R-groups in *D*. *melanogaster* F_329_ and L_332_. Dashed ellipse and curved arrow in (B, C) illustrate the collapse from full inflation due to replacement of a bulky hydrophobic residue required to support the regional structure.

The greatest impairment of tRNase Z activity obtained in the Ala scan through the N_dom_T_prox_ region was observed with substitution of bulky hydrophobics spaced three residues apart (F_329_, L_332_) toward the carboxy end of the region (Figs 3 and 4). The most closely corresponding residues in *S*. *cerevisiae* tRNase Z are Y_361_ and F_364_ in α8 (Fig 2A). If the backbone in this region is α-helical or helix-like (in the *D*. *melanogaster* sequence a proline at 330 would be expected to interrupt an α-helical path; Fig 1), these bulky R-groups would point in roughly the same direction, with potential to collaborate in formation of a hydrophobic cluster. Such a local structural subdomain inflated with high hydrophibicity would not be located deep within the protein considering that the flexible linker spans the enzyme surface (Fig 2B). Based on the recent structure 5MTZ [11], the best candidate hydrophobic partners are I_391_ and I_393_ in β13 of *S*. *cerevisiae* tRNase Z (Fig 9A). α8 in N_dom_T_prox_ is the last homology block at the carboxy end of the amino domain before the start of the flexible linker. β13 is on the carboxy side of the N-T hydrophilic patch that marks the amino boundary of the flexible linker, corresponding to the flexible region sensitive to trypsin (K_348_KTKL) in *D*. *melanogaster* tRNase Z which gives rise to the C_dom_1 species (Figs 5 and 8, cf [14]). Corresponding hydrophilic residues in *S*. *cerevisiae* tRNase Z (E_387_KDN; blue in Fig 9) are in a short helical element with R-groups facing solvent. Bulky hydrophobic pairing partners for *D*. *melanogaster* F_329_ and L_332_ in the N-T region of the flexible linker cannot be identified due to imperfect alignment (Fig 2A).

Internal subdomains are apparently created by juxtaposition of several bulky hydrophobic R groups shielded from solvent, producing a micellar spherule inflated like a beach ball (Fig 9A). Substitution of either of the identified bulky hydrophobic R groups in N_dom_T_prox_ with the single methyl group of alanine (white in Fig 9B and 9C) leads to hydrophobicity collapse (illustrated with dashed ellipses and arrows). The Y_361_ side chain -OH also makes a polar contact with the I_322_ backbone amino group in the β12—α7 loop (not shown); the bulky hydrophobic character of Y_361_ is, however, probably more important than the polarity of its OH group.

### Longer range effects of N_dom_T_prox_ and T_flex_ substitutions on the skeleton of twisted β sheets flanking the flexible arm in the amino domain

**Fig 10:**
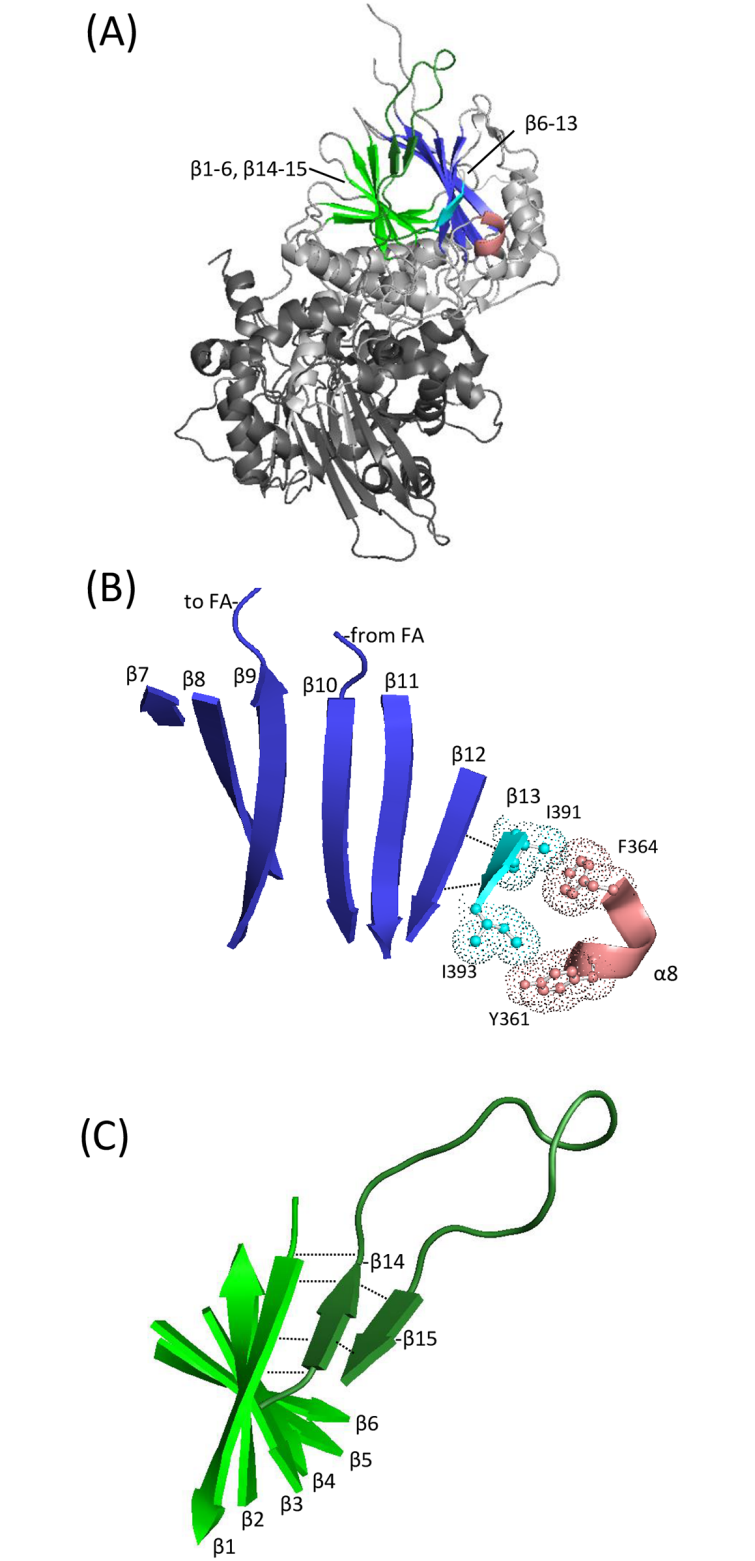
Linker interactions with two skeletal β-twisted sheets in the amino domain of tRNase Z^L^. As illustrated using the crystal structure of *S*. *cerevisiae* Trz1 [11], short β strands in the flexible linker are incorporated by polar backbone contacts into the two β twisted sheets which provide the structural core of the amino domain tRNase Z^L^. (A) Overview of the *S*. *cerevisiae* Trz1 structure (PDB 5MTZ) with the two β twisted sheets in the amino domain highlighted. (B) Isolated view of the β twisted sheet (β7-β13) rotated for optimal viewing of the β strands. The flexible arm is extruded from the body of tRNase Z between β9 (ascending) and β10 (descending). In the linker, residue H_392_ in β13 (cyan) forms polar backbone contacts (dashed lines) with H_315_ and I_317_ in β12, the neighboring parallel strand. Hydrophobic interactions between bulky hydrophobic residues in α8 of N_dom_T_prox_ and β13 of T_flex_, shown in Fig 9, are also presented here. (C) View of the second β twisted sheet (β14-15-1-6), showing antiparallel polar backbone contacts between β14, β15, and β1 (dashed lines). N_415_ in β15 forms backbone polar contacts with T_401_ in β14. Two residues in β14, V_400_ and F_402_, form backbone polar contacts with F_4_ and F_2_ in β1, respectively.

Substitutions in both N_dom_T_prox_ and T_flex_ regions exert their effects through interactions with the skeleton of two twisted β sheets that organize the amino domain (Fig 10). Fig 10A shows the full structure of *S*. *cerevisiae* tRNase Z (5MTZ) with the amino domain light grey, carboxy domain dark grey, twisted β sheet 15, 14, 1–6 green and 13–7 blue. The hydrophobicity collapse in α8/β13 could thus be transmitted into the twisted β sheet in the amino domain on the carboxy side of the flexible arm, as suggested by the enlarged view in Fig 10B.

β13 is a member on one edge of a 7-stranded β sheet, in which H_392_ makes backbone H-bonds with the carboxy group of H_315_ and the amino group of H_317_ in β12 (Fig 10A and 10B). The first four strands from β13 (13/12/11/10) are parallel; the last two strands (10/9/8/7) are antiparallel, and β9,10 ascend to and descend from the flexible arm, respectively. The collapse (deflation) of the α8—β13 spherule arising from substitution of the specific bulky hydrophobic residues in N_dom_T_prox_ with Ala (Fig 9B and 9C) damages the overall fold of tRNase Z, explaining the 2,700-fold and 700-fold impairment of tRNase Z activity (Figs 3 and 4). This also reduces local flexibility (Fig 5) by occluding the N-T site that produces the C_dom_1 family of spots relative to T_flex_, which produces C_dom_2. In some ways, these long-range effects of changes in internal subdomain hydrophobicity resemble those of the L187A substitution at the flexible arm-hand boundary in the ascending stalk of *D*. *melanogaster* tRNase Z, which causes a close to 100-fold impairment in enzyme activity due to increased *K*_*M*_ [14], accompanied by increased flexibility [13].

T_flex_ coincides with a short β strand (β15), one of two short antiparallel β strands in the linker (β15–14) which join a twisted sheet (β1—β6) on the amino side of the flexible arm through backbone H-bonds between β14—β1 (Fig 10C). Concerning the strongest impairment observed in the region with the R382A substitution (Figs 6 and 7), ionized residues on the surface of the protein such as E_419_ and D_422_ in the *S*. *cerevisiae* tRNase Z β15-α9 loop face the polar solvent as expected for T_flex_. Replacement with a small hydrophobic residue could lead to structural eversion in which the substituted residue buries itself in a partially exposed hydrophobic patch, like the effects of the HbS substitution on hemoglobin structure and function.

The reduced flexibility at the T_flex_ site arising from the N378A substitution (Fig 8) is interpretable in a general way (Fig 10C). The short antiparallel β strands β14–15 in the *S*. *cerevisiae* tRNase Z linker are joined through β1 to an 8-stranded twisted sheet in the order β15-14-1-2-3-4-5-6 (β15-14-1-2-3 are antiparallel and β3-4-5-6 are parallel). The flexible linker clearly associates here with the twisted β sheet which forms half the skeleton of the amino domain, on the amino side of the flexible arm (Fig 10C).

Based on the alignment in Fig 2A, N_415_ in *S*. *cerevisae* tRNase Z is the most similar residue in position and identity to N_378_ in *D*. *melanogaster* tRNase Z. Replacement of N_415_ in β15 with a small hydrophobic residue would locally reduce linker flexibility by strengthening skeletal architecture of the amino domain preceding the flexible arm. The reduced linker flexibility arising from the N378A substitution in *D*. *melanogaster* tRNase Z (Figs 6 and 7) could thus improve catalytic efficiency by stiffening the skeleton of β structure on the amino side of the flexible arm. Also noteworthy in this regard, the conservative substitution Leu423Phe in *H*. *sapiens* tRNase Z^L^ (ELAC2) associated with mitochondrially based cardiac hypertrophy [21] is located at the start of β15.

## Conclusion

A biochemical exploration of little-understood regions of *D*. *melanogaster* tRNase Z through Ala scanning mutagenesis followed by processing kinetics was aided by analysis of flexibility using limited proteolysis and two-dimensional protein electrophoresis. This approach, informed by interpretation of a recent crystal structure of the *S*. *cerevisiae* homolog, uncovered a previously unknown hydrophobic subdomain formed across the amino domain—linker boundary, leading us to suggest that peripheral substitutions affect the skeleton of twisted β sheets in the amino domain on both sides of the flexible arm.
